# Shared Decision-Making in Chronic Patients with Polypharmacy: An Interventional Study for Assessing Medication Appropriateness

**DOI:** 10.3390/jcm8060904

**Published:** 2019-06-24

**Authors:** Valle Coronado-Vázquez, Juan Gómez-Salgado, Javier Cerezo-Espinosa de los Monteros, Diego Ayuso-Murillo, Carlos Ruiz-Frutos

**Affiliations:** 1 Research Network on Preventive Activities and Health Promotion (Aragonese Research Group in Primary Care b21-17r), General Directorate of Health Assistance, Aragonese Health Service, 50017 Zaragoza, Spain; mvcoronado@msn.com; 2 Department of Sociology, Social Work and Public Health, University of Huelva, 21007 Huelva, Spain; frutos@uhu.es; 3 Safety and Health Posgrade Program, Universidad Espíritu Santo, Guayaquil 091650, Ecuador; 4 Andalusian Agency for Healthcare Quality, 41092 Seville, Spain; juanj.cerezo.sspa@juntadeandalucia.es; 5 General Secretary, Consejo General de Enfermería, 28023 Madrid, Spain; d.ayuso@consejogeneralenfermeria.org

**Keywords:** inappropriate medications, decision-making support tools, polypharmacy

## Abstract

Potentially inappropriate medications are associated with polypharmacy and polypathology. Some interventions such as pharmacotherapy reviews have been designed to reduce the prescribing of inappropriate medications. The objective of this study is to evaluate how effective a decision-making support tool is for determining medication appropriateness in patients with one or more chronic diseases (hypertension, dyslipidaemia, and/or diabetes) and polypharmacy in the primary care setting. For this, a quasi-experimental study (randomised, controlled and multicentre) has been developed. The study compares an intervention group, which assesses medication appropriateness by applying a decision support tool, with a control group that follows the usual clinical practice. The intervention included a decision support tool in paper format, where participants were informed about polypharmacy, inappropriate medications, associated problems and available alternatives, as well as shared decision-making. This is an informative guide aimed at helping patients with decision-making by providing them with information about the secondary risks associated with inappropriate medications in their treatment, according to the Beers and START/STOPP criteria. The outcome measure was the proportion of medication appropriateness. The proportion of patients who confirmed medication appropriateness after six months of follow-up is greater in the intervention group (32.5%) than in the control group (27.9%) *p* = 0.008. The probability of medication appropriateness, which was calculated by the proportion of drugs withdrawn or replaced according to the STOPP/Beers criteria and those initiated according to the START criteria, was 2.8 times higher in the intervention group than in the control group (OR = 2.8; 95% CI 1.3–6.1) *p* = 0.008. In patients with good adherence to the treatment, the percentage of appropriateness was 62.1% in the shared decision-making group versus 37.9% in the control group (*p* = 0.005). The use of a decision-making support tool in patients with potentially inappropriate medications increases the percentage of medication appropriateness when compared to the usual clinical practice.

## 1. Introduction

Polypharmacy is frequent in polypathological patients, constituting a health problem, as it increases morbidity, risk of falls, adverse effects, hospital admissions, and utilisation of health resources [1]. Patients with chronic diseases and polypharmacy are defined as having one or more chronic diseases (hypertension, dyslipidaemia, and/or diabetes) and having polypharmacy. This term is understood as taking more drugs than is clinically appropriate, although it is often considered that a patient has polypharmacy when taking five or more drugs in a chronic way (for more than six months) [2]. It is estimated that 30% of patients with polypharmacy present potentially severe interactions or avoidable adverse effects [3].

Spain is the second highest country in the world for drug consumption, with the prescription rate of anxiolytics being higher than average for the European Union [4].

Approximately half of the elderly population is prescribed a non-suitable or non-desirable drug, or one that has low therapeutic usefulness, and this is the case more frequently in polypathological and institutionalised patients [5]. In some cases, the prevalence of potentially inappropriate medications can reach 71% in frail elderly people [6].

Following the adequacy criteria put forward by Hanlon [7] and Beers [8], medications are inappropriate when they are potentially harmful and can be avoided because there is an equally effective and less damaging alternative. To evaluate the appropriateness and therapeutic adequacy, there are implicit methods that are based on a clinical assessment of the patient and the administered medication, and explicit methods that are more reliable and based on defined criteria [9]. Among the implicit methods, the one providing the highest validity and reliability is the MAI (Medication Appropriateness Index) [10]. This is a 10-item questionnaire that measures the appropriateness or lack of appropriateness of a medication on a Likert-type scale. However, it has a disadvantage: the long lapse of time its application implies. In addition, it does not measure non-compliance, and it depends on the knowledge and experience of the prescriber [11]. Explicit methods are obtained from a consensus of experts. With these, overprescribing, underprescribing and inappropriate prescribing situations can be detected. Among the possible methods, we will discuss the Beers criteria and the START/STOPP criteria.

The Beers criteria are the most widely used criteria to evaluate potentially inappropriate prescribing in people over the age of 65 [12]. The latest update to these criteria was made in 2012, collecting 53 drugs that are divided into three categories. Three lists of inappropriate medications for the elderly are included. One of them considers the patient’s comorbidities; the second is independent of the diagnosis; and the third includes medications that must be used with caution in people over the age of 75. The main drawback of these criteria is that they do not assess the clinical aspects of the patient.

Other tools are the START criteria (Screening Tool to Alert Doctors to Right Treatment) and the STOPP criteria (Screening Tool of Older Persons’ Prescriptions). They have been designed by an expert panel of the European Union Geriatric Medicine Society. They consist of two instruments, one with 22 indicators of prescription of drugs for prevalent diseases (START) and another with a list of 65 clinically relevant criteria for potentially inappropriate prescribing (STOPP). They are organised by physiological systems and can be applied in a short period of time. Both showed high reproducibility, as well as high reliability and appropriate inter-observer concordance. They have been validated and adapted to the Spanish context [13]. STOPP criteria are a validated list of 65 potentially inappropriate medications (PIMs) for the elderly. They consider drug interactions and drug‒disease interactions. Each criterion includes an explanation of why the prescription is considered inappropriate. The START criteria are made up of a list of 22 items that reflect the medication prescription omission that would apply to the elderly patient under specific clinical situations [14]. At present, STOPP/START is the only set of explicit geriatric prescribing criteria that has shown tangible clinical benefits in the elderly when tested in clinical trials [15].

The prevalence of potentially inappropriate prescriptions is 36% in primary care, 54% in geriatrics consultation, and 50% in institutionalised patients. The inappropriate prescribing of drugs is related to morbidity [16], mortality [17], and the overuse of healthcare resources such as hospital readmissions [11].

With active monitoring of prescriptions, polypharmacy can be more than halved [18]. On the other hand, the participation of patients in decision-making appears in guidelines and developing clinical practice due to their role in the prevention of safety-related incidents regarding treatment. The Agency for Healthcare Research and Quality (AHRQ) of the United States offers tools that help professionals and patients promote safer care in the Primary Care setting. It includes resources aimed at engaging patients in their own care such as leaflets providing advice on the use of gloves before, during and after a medical consultation, guidelines for the safe use of anticoagulants, assistance in preparing outpatient surgery, and materials to improve physician‒patient communication [19].

Shared decision-making (SDM) is a form of physician‒patient relationship applicable to any clinical event (whether diagnostic, therapeutic or preventive), that has been studied in chronic diseases, cancer and palliative care [20]. It is integrated into so-called “patient-centred care,” which involves focusing healthcare on the patients instead of on the assisting professionals [21]. It is considered a process through which it is possible for professionals and patients to make decisions based on the best evidence, considering their values and preferences [22]. It is widely used when the benefits of the intervention are not expected or these are outweighed by the risks. It fits into the deliberative model, including three elements [23]:

- Exchange of information between the physician and the patient.
- Deliberation on the different options.
- Agreed decision.

Decision Support Tools (DSTs) describe in sufficient detail and following the latest evidence the different options available, as well as their risks and benefits [24], with the aim of encouraging patient participation in decisions that affect their health, including their values and preferences.

In this multicentre study, the work hypothesis was that, compared to the usual clinical practice, a shared decision-making intervention is effective at adapting the drug regimen in elderly patients with chronic illnesses and with polypharmacy.

The main objective of this study was to determine the effectiveness of a shared decision-making intervention for medication appropriateness in patients with chronic diseases and polypharmacy.

The difference in medication appropriateness between the intervention group and the control was assessed.

The factors associated with the medication appropriateness were analysed, as well as the differences between the intervention and the control group in the first visit and again after six months in terms of:

- STOPP and Beers withdrawn or substituted drugs and START initiated drugs.
- Percentage of benzodiazepines, non-steroidal anti-inflammatory drugs (NSAIDs) and duplicates that are withdrawn or substituted.

## 2. Experimental Section

### 2.1. Design of the Study

A randomised, multicentre quasi-experimental study was conducted on elderly patients with polypharmacy and inappropriate medications in their treatment.

The study compares an intervention group, which performs medication appropriateness by applying a decision support tool, with a control group that follows the usual clinical practice.

The patients were recruited by family physicians from health centres in Aragon and Andalusia, Spain.

Before starting the study, this project received a favourable report from the Clinical Research Ethics Committee of Aragon, with code number P115/0306 adopted on 21 December 2015.

### 2.2. Participants

Teams of family physicians and nurses from 11 health centres were invited to participate. Twenty-two of them agreed to participate.

Patients were included in the study if they met the following criteria: aged hi 65 or over, one or more chronic diseases (hypertension, dyslipidaemia and/or diabetes), with polypharmacy (taking five or more drugs for more than six months), and taking, at least, one potentially inappropriate medication according to the Beers and/or STOPP criteria, or to a START criterion.

Those patients who were unable to make decisions due to cognitive impairment or mental retardation, those receiving palliative care or the institutionalised were excluded.

### 2.3. Randomisation and Masking

Physicians who agreed to participate in the study were randomised and assigned to the intervention or the control group. A block-randomisation procedure was carried out to ensure the equal size of the groups.

As the intervention affects professionals’ way of communicating, to avoid contamination, no randomisation of the patients was done.

The professionals selected the participants from the listings of patients with polypharmacy from their patients’ portfolio.

### 2.4. Intervention

The intervention design followed the model by Elwyn [25], which transplants the concept of shared decisions to clinical practice with an easy-to-remember tool that serves as a guide for decision-making. The model has three key steps, namely: “choice talk,” “option talk” and “decision talk.”

Applied to medication appropriateness, the intervention included a decision support tool (DST) in paper format, where participants were informed about polypharmacy, inappropriate medications, associated problems and available alternatives, as well as shared decision-making. The DST was developed by physicians and nurses of the research team. This is an informative guide aimed at helping patients with decision-making by providing them with information about the secondary risks associated with inappropriate medications in their treatments according to the Beers and START/STOPP criteria. It allows for comparing the positive and negative aspects of the different treatment options according to these criteria. Once inappropriate medications are detected, the patient is informed and given alternatives, such as their suspension or substitution with other medications. With DSTs, patients are assisted in clarifying the tool’s values and discussing them with the professional involved (Supplementary brochures 1 and 2).

#### 2.4.1. Development of the Intervention

Physicians who participated in the intervention group received information on the designed DST, as well as a link to a video about the shared decision-making process, which was available on the web.

Once the patients were selected, the family physicians reported the inappropriateness found in their medication according to the Beers and START/STOPP criteria, and the possible alternatives they had, agreeing on the changes to be made in the treatment through a deliberative process.

#### 2.4.2. Usual Clinical Care

In the control group, family physicians discussed with patients the inappropriate medications. No DST was used in this group, and care was not standardised.

### 2.5. Data Collection

The selected patients were assessed by family physicians to verify that they met the inclusion criteria. They were informed about the study and gave informed consent to participate in it.

From the electronic medical record, the personal data and medical record of the patients were collected, including the updated treatment. The nurses performed a functional, mental and social assessment, also determining the level of adherence to the treatment. The family physician analysed the adequacy of the treatment for each patient following the Beers, START and STOPP criteria through the data collected in the electronic medical records, which were verified during the visit.

The patients were summoned to the doctor’s office to carry out the medication appropriateness survey using the DST in the intervention group or the usual clinical practice in the control group.

After six months, the family physician contacted the participants again, either through regular visits to follow up chronic patients or by telephone. In this second meeting with the family physician, the medication was re-checked, making a new intervention in case of inappropriate medications.

### 2.6. Outcomes Measures

The main variable was the difference between groups regarding the proportion of medication appropriateness, understood as the withdrawal or substitution of drugs according to the STOPP/Beers criteria and the initiation of drugs following the START criteria.

The secondary variables were:

- The average number of drugs, according to the STOPP and Beers criteria, that had been withdrawn and substituted in the first visit or after six months of follow-up.
- Percentage of benzodiazepines, nonsteroidal anti-inflammatory drugs (NSAIDs) and duplicated medications that were withdrawn or substituted in the first visit and after six months.
- Types of drugs withdrawn or substituted, according to the STOPP/Beers criteria, and drugs initiated according to the START criteria.

### 2.7. Statistical Analysis

A sample size of 137 patients led to a 14% difference in medication appropriateness between the control and the intervention groups (START/STOPP and Beers criteria), with a power of 80%, a confidence level of 95% and an expected proportion of 10% losses.

An analysis was made by intention to treat.

Proportions, means and standard deviations were used to describe the patients in the study groups. The bivariate analysis was performed with the Chi-squared test for categorical variables and Student’s *t*-test for continuous variables. A subgroup analysis was done, and the OR was calculated to determine the association between the intervention and the medication appropriateness. The NNT and the 95% confidence interval were determined. 

## 3. Results

**Figure 1:**
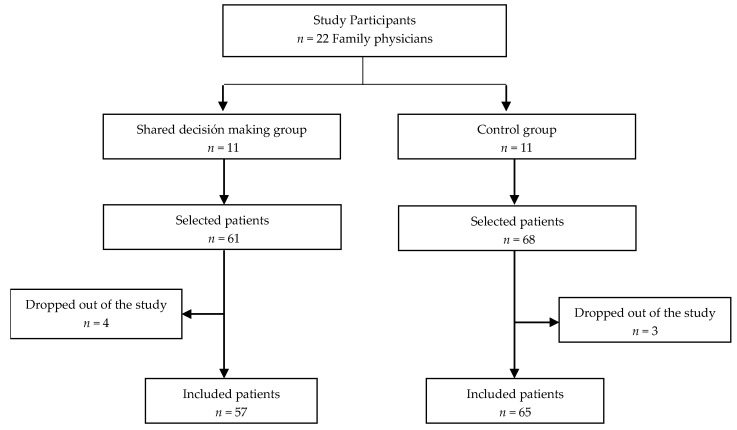
Participant flowchart.

From November 2015 to March 2016, 22 family physicians from different health centres in Aragon and Andalusia were contacted and randomised to the intervention or control groups (Figure 1). At the beginning of the field work, there were seven physicians who abandoned the study (four in the intervention group and three in the control group).

For each medical portfolio, patients were selected among those who met the inclusion criteria. These were recruited in the doctor’s office. For the follow-up, contact was made either through scheduled consultations or by telephone.

Three patients were not monitored: two did not wish to continue and one was withdrawn by admission to an institution.

The sample consisted of 122 patients (57 in the shared decision-making group and 65 in the usual clinical practice group).

Table 1 summarises the characteristics of the participants by groups. The mean age was 79.9 years (SD 6.4) and 78 (63.9%) were women; 70.2% had completed primary education.

On average, they presented with six chronic diseases (SD 1.8): 90.2% were hypertensive, 40.2% were diabetic, and 17.2% had kidney failure. The mean of chronically prescribed drugs was nine (SD 2.9). There were no significant differences regarding the baseline characteristics among the study groups, except for the number of chronic diseases (Table 1).

A total of 182 potentially inappropriate medications was found when following the STOPP criteria, and 95 according to the Beers criteria. The most frequent were benzodiazepines (BDZ) and non-benzodiazepine hypnotics, NSAIDs, proton pump inhibitors (PPIs) and duplicity. Following the START criteria, treatment had to be started for 19 drugs (Table 2). According to the STOPP criteria, 86.1% of patients in the sample had at least one potentially inappropriate medication, and 64.8% according to the Beers criteria. In 13.9%, treatment with at least one drug had to be started, according to the START criteria.

### Effect of the Shared Decision-Making Intervention on Medication Appropriateness

The proportion of patients whose medication was adapted after six months of follow-up was higher in the intervention group (32.5%) than in the control group (27.9%), *p* = 0.008.

The probability of medication appropriateness was 2.8 times higher in the intervention group than in the control group (OR = 2.8; CI 95% 1.3 to 6.1), *p* = 0.008.

The NNT was 4 (CI 95% 2.6 to 16.7), which implies that, to achieve the appropriateness of an additional drug, it was necessary to perform the intervention on four patients.

The medication appropriateness was performed among both sexes, depending on the type of adherence to the treatment, the use of benzodiazepines and non-steroidal anti-inflammatory drugs, and the level of education (Table 3).

In patients with good adherence to the treatment, the percentage of appropriateness was 62.1% in the shared decision-making group, as compared to 37.9% in the control group (*p* = 0.005). The medication appropriateness was also greater in the intervention group for those patients who did not take benzodiazepines (BDZ) (54.5% vs. 45.5%, *p* = 0.019) or non-steroidal anti-inflammatory drugs (67.4% vs. 32.6%, *p* = 0.001), and in the group of educated patients (61% vs. 39%, *p* = 0.001). In case of duplication, the medication was withdrawn in 66.7% of the patients in the intervention group against non-withdrawal in the control group (*p* = 0.035). The average of inappropriate medications withdrawn after the follow-up was higher in the intervention group than in the control group (x = 1 (SD 0.9) vs. x = 0.66 (SD 0.7)), mean difference 0.34 (CI 95%: 0.01–0.66), *p* = 0.04 (Table 4).

**Figure 2:**
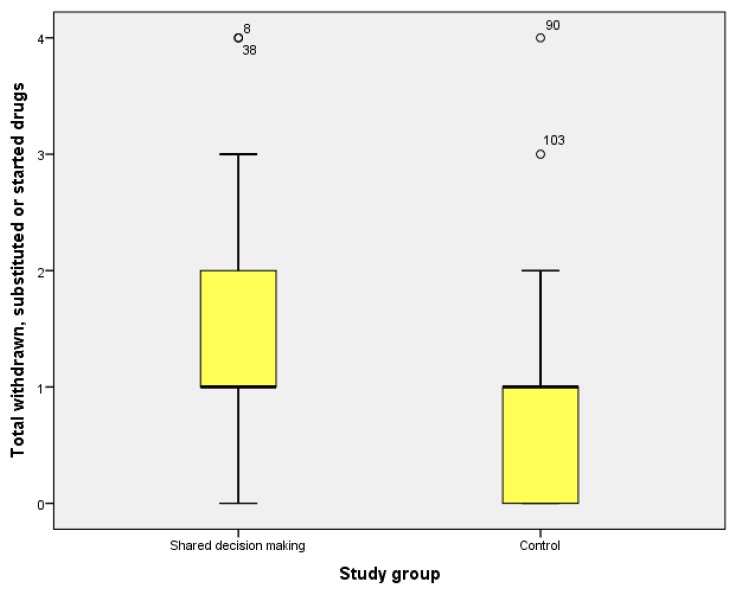
Drugs withdrawn or started, by groups.

In patients with renal failure, NSAIDs were withdrawn in 75% of the control group (*p* = 0.03) and BDZ in 83.3% of the intervention group (*p* = 0.22) (Figure 2).

## 4. Discussion.

In elderly patients with polypharmacy and inappropriate medications in their treatments, the use of a decision-making tool to adapt the drug regimen, compared with the usual clinical practice, is associated with a greater number of withdrawn and substituted drugs, as well as with a higher proportion of medication appropriateness. The inappropriately prescribed medications found most commonly were long-term BDZ, NSAIDs, and PPIs, in addition to medication duplicity. These findings are in line with the study by Rivas-Cobas on polypathological patients, which describes the taking of BDZ as the most common criterion of inappropriate medication [26].

In this study, medication appropriateness was 2.8 times more likely when using a decision support tool rather than the usual clinical practice. The proportion of patients with withdrawn or started treatment was greater in this group. Although this difference is not as high as expected, the resulting NNT indicates that the adequacy of medication when using a decision support tool is better than in usual clinical practice. The effectiveness of the decision support tool could be improved through training sessions aimed at health professionals’ awareness of the problem, dissemination of the tool and training on its use.

Different interventions have been proposed to reduce potentially inappropriate prescriptions. When deprescribing, the most effective interventions are related to a medication review by multidisciplinary teams of professionals [27]. In some cases, medication reviews are made by a clinical pharmacologist, resulting in a discontinuation of inappropriate drugs in up to 69% of the patents [28]. In primary care, there has also been a reduction in the number of patients with potentially inappropriate medications in their treatment after a medication review by a pharmacologist [29].

The development of a web application that evaluates inappropriate medications and proposes recommendations to adapt the treatment improves shared decision-making and reduces errors of medication reconciliation but does not modify the prescription [30]. In other cases, computerised alert systems reduced inappropriate medications [31].

The information sessions carried out by general practitioners and pharmacists showed no difference in the prevalence of inappropriate medications [32], although continued training performed by general practitioners did lead to a reduction of 10.3% in potentially inappropriate medications in older patients [33].

As in other studies [34,35], the inappropriate medications most frequently found in this study were long-term benzodiazepines and NSAIDs such as chronic analgesics for osteoarthritis. Benzodiazepines have negative effects at the cognitive level and on motor functions, making falls more likely and not contributing any positive effects in the long term [36]. However, in our study, medication appropriateness was more common in patients who were not prescribed benzodiazepines in their treatment, probably due to the complexity of the withdrawal or substitution of these drugs.

In patients with renal failure, the withdrawal of NSAIDs was higher in the control group than in the intervention group. Despite the adverse effects associated with the use of NSAIDs in renal failure, these drugs are often still used in elderly patients with renal diseases [37]. In a review, it was found that the prevalence of inappropriate medications in general population patients with renal failure was between 1% and 37%, with antidiabetics, renin‒angiotensin axis-blocking drugs, and uricosuric drugs involved most frequently [38].

With the designed tool, better results were obtained in terms of the medication appropriateness of patients with good adherence to the treatment. It should be considered that some studies have described an association between the number of inappropriate medications and patients with a poor treatment adherence [39], so interventions designed to adapt drugs should have an impact on the latter group. The results were also more favourable with the use of HATD in the group of patients with an education, but not in the uneducated group. It is for this reason that, during the design of these tools, it is important to assess the population at which they are aimed, with the objective of increasing the profitability and improving the results of their application.

### 4.1. Implications for the Practice and Future Research

The decision support tool designed for this study and applied by family physicians increases the percentage of patients for whom medication appropriateness is achieved. In future research, the outcome of this intervention could be analysed considering the long-term reduction of potentially inappropriate medications, valuing the development of tools that are adapted to the level of knowledge of patients and, further on, their development in web format.

### 4.2. Study Limitations

Although the sample size initially calculated was not reached, differences in medication appropriateness were found between the two groups of the selected sample. A larger sample would be needed to assess the effect that some factors have on the withdrawal of inappropriate medications using DST, as well as to determine the outcomes on clinical variables such as reducing drug-related problems.

A randomisation was carried out regarding professional staff to reduce the risk of contamination of the study groups and to control possible resulting bias.

## 5. Conclusions

The use of a decision support tool in patients with polypharmacy identifies inappropriate medications and enhances the prevalence of appropriate prescribing compared to the usual clinical practice. The proportion of patients whose drug regimen was successfully adapted using the decision support tool was higher among those with better adherence to the treatment and those who do not take benzodiazepines or non-steroidal anti-inflammatory drugs.

## Figures and Tables

**Table 1:** Characteristics of the study patients.

Patients’ characteristics	Shared Decisions (*n* = 57)	Control (*n* = 65)	*p*
**Sex***n* (%)	0.56		
Female	38 (66.7)	40 (61.5)	
Male	19 (33.3)	25 (38.5)	
**Age x** (SD)	78.9 (0.94)	79.9 (0.73)	0.38
**Level of education***n* (%)			0.36
None	14 (25)	17 (29.3)	
Primary school	39 (69.6)	41 (70.7)	
Secondary school	2 (3.6)	0	
University	1 (1.8)	0	
**Chronic diseases**x (SD)	5.7 (0.25)	6.45 (0.21)	0.03
**Hypertension***n* (%)	52 (91.2)	58 (89.2)	0.71
**Diabetes***n* (%)	25 (43.9)	24 (36.9)	0.44
**Renal failure *n* (%)**	12 (21.1)	9 (13.8)	0.29
**Liver failure *n* (%)**	2 (3.5)	0	0.12
**Prescribed medications**x (SD)	9.37 (0.39)	9.29 (0.35)	0.89
**Prescribed medication in:**			
Hypertension x (SD)	9.54 (2.9)	9.34 (2.9)	0.73
Diabetes x (SD)	10.36 (3)	10.04 (3.2)	0.72
Renal failure x (SD)	9.5 (2.5)	11.2 (2.7)	0.74
**DADL***n* (%)			0.43
Independent	33(57.9)	28 (52.8)	
Low	22 (38.6)	19 (35.8)	
Moderate	1 (1.8)	2 (3.8)	
High	1 (1.8)	1 (1.9)	
Total	0	3 (2.7)	

DADL: Dependence in activities of daily living.

**Table 2:** Types of drugs according to the STOPP, Beers and START criteria prescribed for each group patient.

Drugs	Intervention (*n* = 57)	Control (*n* = 65)	*p*
**STOPP criteria drugs *n* (%)**			
Medium‒long-term BDZ	22 (44)	28 (56)	0.82
PPIs	20 (44.4)	25 (55.6)	0.77
NSAIDs	3 (15)	17 (85)	0.005
Duplicities	13 (68.4)	6 (31.6)	0.013
**Other**			0.47
A1 Digoxin	0	1	
A2 Loop diuretics	3 (37.5)	5 (62.5)	
Opioids	2 (28.6)	5 (71.4)	
A4 Thiazide with gout	1 (25)	3 (75)	
E8 Colchicine	1 (50)	1 (50)	
A7 Diltiazem	0	1 (100)	
A12 (ASA > 150)	2 (28.6)	5 (71.4)	
H4 Vasodilators	2 (100)	0	
B5 TCAs	2 (100)	0	
F Tamsulosin	1 (50)	1	
B12 SSRI	0	1 (100)	
A10 Dipyridamole	0	1 (100)	
H3 Antihistamine	1 (100)	0	
**START criteria drugs *n* (%)**			
Statins	5 (62.5)	3 (37.5)	0.72
ACEIs	2 (100)	0	0.2
Metformin	1 (50)	1	0.78
**Beers criteria drugs *n* (%)**			
BDZ+ Non-BDZ hypnotics	32 (50)	32	0.14
NSAIDs	0	7 (100)	0.01
Anticholinergics	5 (62.5)	3 (37.5)	0.32
**Other**			0.34
Alpha-1 blockers	1 (50)	1	
Antipsychotics	2 (100)	0	
Antiarrhythmics	2 (40)	3 (60)	

BDZ: Benzodiazepines; PPIs: Proton Pump Inhibitors; NSAIDs: Nonsteroidal anti-inflammatory drugs; ASA: Acetylsalicylic acid. TCAs: Tricyclic antidepressants. SSRIs: Selective serotonin reuptake inhibitors. ACEIs: Angiotensin-converting enzyme inhibitors.

**Table 3:** Effect of shared decision-making on medication appropriateness by subgroups.

Medication appropriateness	Intervention *n* (%)	Control *n* (%)	*X^2^*	*p*
**Medication appropriateness**	*n* = 57	*n* = 65		
**Sex**				
Male	28 (57.1)	21 (42.9)	3.74	0.053
Female	15 (53.6)	13 (46.4)	3.38	0.066
**Adherence to treatment**				
Good	41 (62.1)	25 (37.9)	7.9	0.005
Bad	2 (18.2)	9 (81.8)	0.01	0.91
**Treatment with BDZ**				
Yes	16 (51.6)	15 (48.4)	1.9	0.16
No	18 (54.5)	15 (45.5)	5.4	0.019
**Treatment with NSAIDs**				
Yes	3 (16.7)	15 (83.3)	0.39	0.53
No	31 (67.4)	15 (32.6)	12.9	0.0001
**Level of education**				
None	7 (38.9)	11 (61.1)	0.68	0.409
Educated	36 (61)	23 (39)	11	0.0001

BDZ: Benzodiazepines; NSAIDs: Nonsteroidal anti-inflammatory drugs.

**Table 4:** Effect of the intervention on the number of inappropriate medications withdrawn at the first consultation and after six months.

Medications withdrawn	Intervention *n* = 57	Control *n* = 65	Means difference (CI 95%)	*p*
**STOPP medications** **x** **(SD)**				
Baseline	1.32 (0.11)	1.65 (0.12)		
Withdrawn at 1st consult.	0.63 (0.09)	0.49 (0.08)	−0.13 (−0.39 to 0.11)	0.27
Withdrawn after 6 months	0.11 (0.4)	0.05 (0.2)	−0.05 (−0.18 to 0.06)	0.34
Total withdrawn	1 (0.94)	0.66 (0.87)	−0.33 (−0.66 to −0.013)	0.04
**Started drugs** **x** **(SD)**				
Started at 1st consult.	0.19 (0.44)	0.12 (0.37)	0.07 (−0.07 to 0.21)	0.34
**Total withdrawn and started drugs**				
**x (SD)**	1.19 (1)	0.78 (0.90)	0.408 (0.06 to 0.75)	0.02

## Supplementary Materials

The following are available online at https://www.mdpi.com/2077-0383/8/6/904/s1, Information for patients (Supplementary brochures 1 and 2).
